# Selective excitation of hyperbolic phonon polaritons-induced broadband absorption via *α*-MoO_3_ square pyramid arrays

**DOI:** 10.1186/s11671-023-03825-5

**Published:** 2023-03-12

**Authors:** Chui Pian, Tian Sang, Shi Li, Chaoyu Yang, Xianghu Zhang

**Affiliations:** 1 Department of Photoelectric Information Science and Engineering, School of Science, Jiangnan University, Wuxi, 214122 China; 2 Jiangsu Provincial Research Center of Light Industrial Optoelectronic Engineering and Technology, Jiangnan University, Wuxi, 214122 China

**Keywords:** Selective broadband absorption, Square pyramid arrays, Hyperbolic phonon polaritons, Effective medium theory, Fabry–Pérot resonance

## Abstract

Optical anisotropy of *α*-MoO_3_ in its reststrahlen (RS) bands provides exciting opportunities for constructing the polarization-dependent devices. However, achieving broadband anisotropic absorptions through the same *α*-MoO_3_ arrays is still challenging. In this study, we demonstrate that selective broadband absorption can be achieved by using the same *α*-MoO_3_ square pyramid arrays (SPAs). For both the *x* and *y* polarizations, the absorption responses of the *α*-MoO_3_ SPAs calculated by using the effective medium theory (EMT) agreed well with those of the FDTD, indicating the excellent selective broadband absorption of the *α*-MoO_3_ SPAs are associated with the resonant hyperbolic phonon polaritons (HPhPs) modes assisted by the anisotropic gradient antireflection (AR) effect of the structure. The near-field distribution of the absorption wavelengths of the *α*-MoO_3_ SPAs shows that the magnetic-field enhancement of the lager absorption wavelength tends to shift to the bottom of the *α*-MoO_3_ SPAs due to the lateral Fabry–Pérot (F–P) resonance, and the electric-field distribution exhibits the ray-like light propagation trails due to the resonance nature of the HPhPs modes. In addition, broadband absorption of the *α*-MoO_3_ SPAs can be maintained if the width of the bottom edge of the *α*-MoO_3_ pyramid is large than 0.8 μm, and excellent anisotropic absorption performances are almost immune to the variations of the thickness of the spacer and the height of the *α*-MoO_3_ pyramid.

## Introduction

Natural van der Waals (vdW) materials have shown great promise to guide the flow of light at the nanoscale from the visible to terahertz region due to their superior physical and chemical properties [1]. In particular, *α*-MoO_3_, as a typically natural vdW semiconductor, has shown the exotic optical performances in the mid-infrared (IR) region due to their excellent hyperbolic phonon polaritons (HPhPs) responses [2]. It is shown that the HPhPs of the *α*-MoO_3_ crystals exhibit strong electromagnetic field confinement, ultraslow group velocities, and long resonance lifetimes [3, 4]. By controlling the ray-like light propagation of the HPhPs in the in-plane of the *α*-MoO_3_ flakes, interesting phenomena such as in-plane nanocavity resonance [5], in-plane Raman spectroscopy [6], in-plane focusing [7, 8], and steerable hyperbolic polaritons [9–11] can be realized. In addition, by using the twisted *α*-MoO_3_ structures [12, 13] or the graphene *α*-MoO_3_ heterostructures [14–16], the in-plane topological transition of the photonic dispersion of phonon polaritons of the *α*-MoO_3_ flakes can be dynamically controlled.

In recent years, the out-of-plane properties of the *α*-MoO_3_ crystals associated with the far-field distributions have attracted significant attention due to their versatile optical performances. By using the highly anisotropic HPhPs modes of the *α*-MoO_3_, polarization converters [17, 18], chirality responses [19, 20], and spinning thermal radiation [21] can be realized in the mid-IR region under the illuminations of the out-of-plane incident wave. Moreover, it is shown that the *α*-MoO_3_-based structures can be functioned as the excellent anisotropic absorbers, and perfect narrowband absorption with single or multiple channels can be achieved at the critical coupling condition [22–24]. Interestingly, to achieve broadband anisotropic absorption in mid-IR, two types of *α*-MoO_3_ trapezoidal nanostructures are constructed to selectively absorb the incident light energy of the *x* and *y* polarizations [25]. However, this approach requires two different *α*-MoO_3_ patch arrays along the* x* or *y* directions, and each patch array can only be functioned as the broadband absorber for the specific polarization state, which limits their further applications due to the additional complexity of the structure. Therefore, achieving broadband anisotropic absorptions through the same *α*-MoO_3_ arrays is crucial and is highly desired.

In this work, selective broadband absorption enhancement is achieved based on the same *α*-MoO_3_ square pyramid arrays (SPAs). The *α*-MoO_3_ SPAs can be equivalent to an anisotropic gradient antireflection (AR) film according to the effective medium theory (EMT), and highly efficient selective broadband absorption can be realized due to the excitations of the resonant HPhPs modes assisted by the anisotropic AR effects of the structure. By studying the near-field distributions of the absorption wavelengths of the *α*-MoO_3_ SPAs, it is shown that the magnetic-field enhancement of the lager absorption wavelength tends to shift to the bottom of the *α*-MoO_3_ SPAs due to the lateral Fabry–Pérot (F–P) resonance, while the electric-field distribution exhibits the ray-like light propagation due to the resonance nature of the HPhPs modes. In addition, good broadband absorption performances of the *α*-MoO_3_ SPAs can be maintained if the width of the bottom edge of the *α*-MoO_3_ pyramid is sufficiently large, and broadband absorption performances are very robust to the variations of the height of the *α*-MoO_3_ pyramid.

## Structural configuration and method

**Fig. 1:**
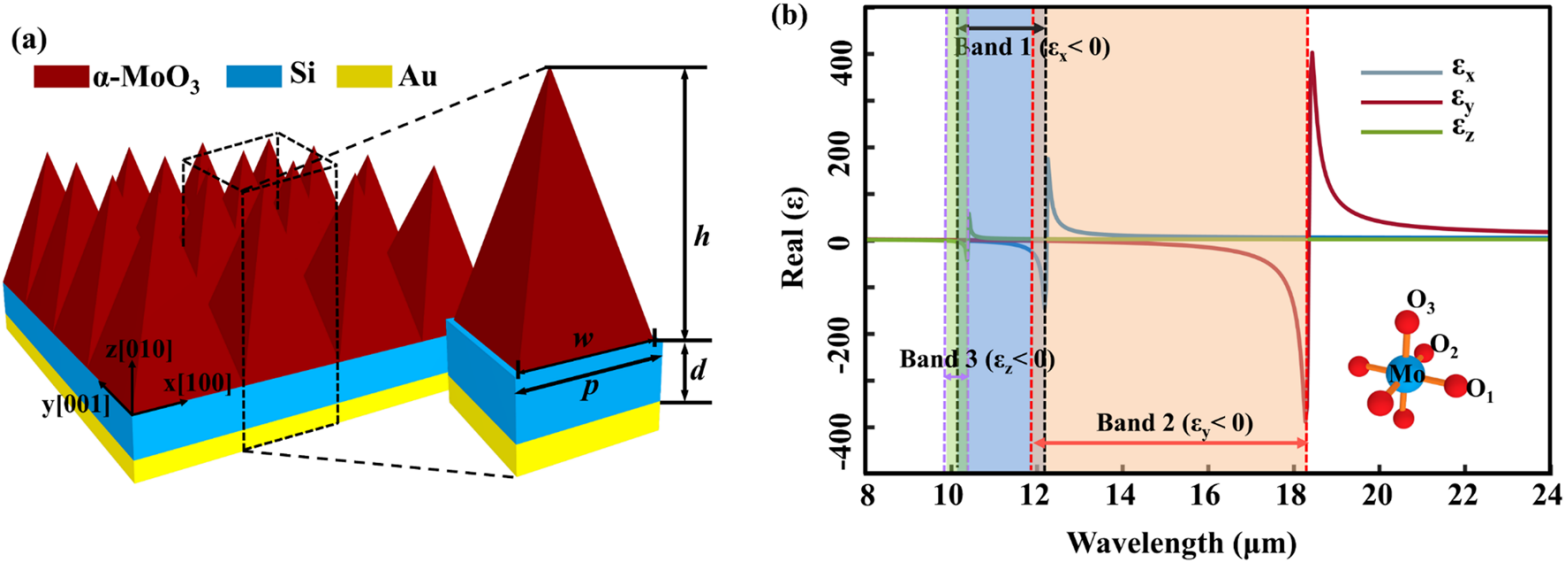
**a** Schematic diagram of the *α*-MoO_3_-based SPAs consisting of *α*-MoO_3_ pyramid arrays and an Au substrate separated by a Si spacer. The unit cell of the structure is enlarged on the right, where *h* and *w* are the height and width of the *α*-MoO_3_ square pyramid, respectively; *d* is the thickness of the spacer, and *P* is the period of the structure. **b** The real part of the dielectric tensor of *α*-MoO_3_ along different crystalline axes, the schematic diagram of atomic orientation of *α*-MoO_3_ is inserted in the figure

Figure 1 shows the schematic diagram of the proposed *α*-MoO_3_-based SPAs and the dielectric tensor of *α*-MoO_3_ along different crystalline axes. As shown in Fig. 1a, the *α*-MoO_3_-based SPAs consist of *α*-MoO_3_ pyramid arrays and an Au substrate separated by a Si spacer. The *α*-MoO_3_ pyramid arrays are periodic along both the *x*-axis (along the [100] crystal direction) and the *y*-axis (along the [001] crystal direction) with the period of *p*. The unit cell of the structure is enlarged on the right, where *h* and *w* are the height and width of the bottom edge of the *α*-MoO_3_ square pyramid, respectively; *d* is the thickness of the spacer. The refractive index of Si is 3.42 in the wavelength region of interest [26]. The Au substrate is a perfect reflector and thick enough to block the light transmission, thus the total absorption of the *α*-MoO_3_ SPAs can be reduced as *A*(*λ*) = 1 − *R*(*λ*), where R(*λ*) denotes the reflection of the structure. In simulation, the absorption response, and the near-field distribution of the *α*-MoO_3_ SPAs are calculated by using the finite-difference time-domain (FDTD) software Lumerical, other features such as the dielectric tensor of the *α*-MoO_3_, the dispersion of the HPhPs modes, and the EMT are calculated by using Matlab codes.

As an anisotropic biaxial vdW crystal, the *α*-MoO_3_ has three oxygen species (O_1−3_) along different crystallographic directions, as shown in the figure inset of Fig. 1b. It is well known that *α*-MoO_3_ has an orthorhombic layered oxide structure composed of distorted and edge-shared MoO_6_ octahedra, wherein each octahedron contains three types of oxygen sites: the terminal O_1_, which is doubly bonded with the Mo atom, the doubly coordinated and asymmetric bridging O_2_, and the triply coordinated and symmetric bridging O_3_ [27, 28]. Such a complex structure leads to a great variety of phonon modes in the mid to far-IR, and the dielectric tensor *ε* = *diag*[*ε*_*x*_,*ε*_*y*_,*ε*_*z*_] of the *α*-MoO_3_ can be described by using the Lorentz model [29–31]:

1 $$\varepsilon_{x} = \varepsilon_{\infty }^{x} \left( {1 + \frac{{\omega_{LO}^{{x}^{2}} - \omega_{TO}^{{x}^{2}} }}{{\omega_{TO}^{{x}^{2}} - \omega^{2} - i\omega \Gamma^{x} }}} \right),$$

2 $$\varepsilon_{{\text{y}}} = \varepsilon_{\infty }^{y} \left( {1 + \frac{{\omega_{LO}^{{y}^{2}} - \omega_{TO}^{{y}^{2}} }}{{\omega_{TO}^{{y}^{2}} - \omega^{2} - i\omega \Gamma^{y} }}} \right),$$

3 $$\varepsilon_{z} = \varepsilon_{\infty }^{z} \left( {1 + \frac{{\omega_{LO}^{{z}^{2}} - \omega_{TO}^{{z}^{2}} }}{{\omega_{TO}^{{z}^{2}} - \omega^{2} - i\omega \Gamma^{z} }}} \right),$$

where *ω* is the angular frequency of the incident wave, and other parameters are: *εx ∞* = 4, *εy ∞* = 5.2, *εz ∞* = 2.4, *ωx LO* = 974 cm^−1^, *ωy LO* = 851 cm^−1^, *ωz LO* = 1010 cm^−1^, *ωx TO* = 818 cm^−1^, *ωy TO* = 545 cm^−1^, *ωz TO* = 962 cm^−1^, *Г*_*x*_ = *Г*_*y*_ = 4 cm^−1^, and *Г*_*z*_ = 2 cm^−1^.

Figure 1b shows the real peart of the dielectric tensor of the *α*-MoO_3_ calculated by using the Lorentz model. As shown in Fig. 1b, the *α*-MoO_3_ exhibits three reststrahlen (RS) bands for three orthogonal crystal axes in the wavelength region of interest. The labeled “bands 1, 2, and 3” represent the three RS bands for *α*-MoO_3_ that originate from the hyperbolic phonon modes along the *x*-, *y*-, and *z*-axes with *ε*_*x*_ < 0 (10.27–12.20 μm), *ε*_*y*_ < 0 (11.76–18.32 μm), and *ε*_*z*_ < 0 (9.91–10.40 μm), respectively.

**Fig. 2:**
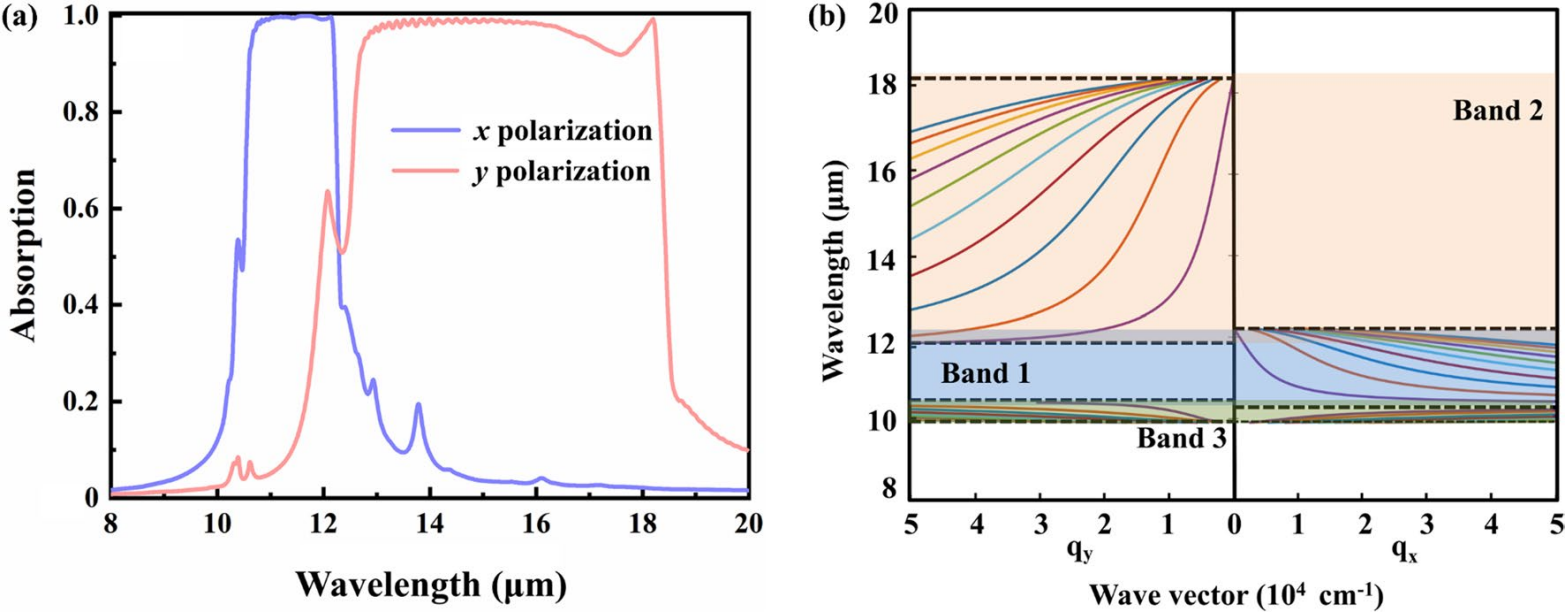
**a** Absorption responses of the *α*-MoO_3_ SPAs under the illuminations of normally incident *x* and *y* polarized waves. The structural parameters are: *h* = 4.8 μm, *d* = 0.9 μm, *p* = *w* = 1.0 μm. **b** Dispersion of the equivalent air/*α*-MoO_3_/Si structure in *xz* (versus *q*_*x*_) and *yz* (versus *q*_*y*_) cross sections, where the thickness of the *α*-MoO_3_ flake is 4.8 μm

Figure 2a shows absorption responses of the *α*-MoO_3_ SPAs under the illuminations of normally incident *x* and *y* polarized waves. The structural parameters are: *h* = 4.8 μm, *d* = 0.9 μm, *p* = *w* = 1.0 μm. As shown in Fig. 2a, the *α*-MoO_3_ SPAs show excellent selective broadband absorption performances in the two major RS bands of band 1 and band 2. For the *x* polarization, highly efficient absorption with average absorption of 99.0% can be achieved within the wavelength range of 10.59–12.19 μm, and the average absorption reaches 92.5% in the whole band 1. In the case of *y* polarization, the average absorption is as large as 97.6% within the wavelength range of 12.67–18.27 μm, and the average absorption is 89.0% in the whole band 2. Note there is a perfect absorption peak at 18.18 μm corresponding to transverse optical (TO) phonon mode indicated in Fig. 1b, which can be excited directly by the optical photons for the *y* polarization. In addition, there are also absorption peaks in band 3 at 10.39 μm for both the *x* and *y* polarizations, which are associated with the excitations of the TO phonon mode at *ωz TO* = 962 cm^−1^.

To better understand the anisotropic broadband absorption performances of the *α*-MoO_3_ SPAs between the RS bands and the optical photon modes, we use the derived analytical dispersion of polaritons propagating in a biaxial slab embedded between two media [32, 33]:

4 $$q = \frac{\rho }{{k_{0} h^{\prime}}}\left[ {\arctan \left( {\frac{{\varepsilon_{1} \rho }}{{\varepsilon_{z} }}} \right) + \arctan \left( {\frac{{\varepsilon_{3} \rho }}{{\varepsilon_{z} }}} \right) + \pi l} \right],\quad l = 0,1,2 \ldots$$

where *ε*_1_ and *ε*_3_ are the permittivities of the superstrate and substrate, respectively; *ρ* = *i* [*ε*_*z*_/(*ε*_*x*_ cos^2^*α* + *ε*_*y*_ sin^2^*α*)]^1/2^, with *α* being the angle between the *x*-axis and the in-plane component vector; *k*_*o*_ = *ω*/*c* is the wavevector in free space, *q* = *k*_*t*_/*k*_*o*_ is the normalized in-plane momentum (*k*_*t*_^2^ = *k*_*x*_^2^ + *k*_*y*_^2^), and *hʹ* is the thickness of the *α*-MoO_3_ flake. For the proposed *α*-MoO_3_-based SPAs, it can be equivalent to the air/*α*-MoO_3_/Si structure, and *ε*_*1*_ = 1, and *ε*_*3*_ = 11.7, *hʹ* = 4.8 μm.

Figure 2b shows the dispersion distribution of the equivalent air/*α*-MoO_3_/Si structure in *xz* (versus *q*_*x*_) and *yz* (versus *q*_*y*_) cross sections by using Eq. (4). As can be seen in Fig. 2b, multiple HPhPs modes can be excited along both the *x* and *y* directions in all the RS bands of the *α*-MoO_3_. Broadband absorption of the *x* polarization is associated with the excitations of the HPhPs modes along the *x* direction with wavevector *q*_*x*_ in band 1. Similarly, broadband absorption of the *y* polarization is associated with the excitations of the HPhPs modes along the *y* direction with wavevector *q*_*y*_ in band 2. Therefore, the excellent anisotropic broadband absorption performances of the *α*-MoO_3_ SPAs are the direct consequence of the excitation of the resonant HPhPs modes. Additional remark is that due to the excitations of the multiple HPhPs modes in band 1 and band 2 indicated in Fig. 2b, slight fluctuations associated with the HPhPs modes are appeared in both the two absorption bands as shown in Fig. 2a.

## Results and discussion

To further the quantitative understanding of the selective broadband absorption performances of the proposed *α*-MoO_3_-based SPAs, the *α*-MoO_3_ pyramid arrays can be equivalent to an effective anisotropic thin film due to the deep-subwavelength structure feature, i.e., the period of the pyramid arrays is much less than the operation wavelengths. According to the EMT [34], the effective dielectric constant of the *α*-MoO_3_ pyramid can be written as:

5 $$\varepsilon_{{{\text{eff}},i}} = (\varepsilon_{i} \cdot \varepsilon_{1} )/[F \cdot \varepsilon_{1} + (1 - F) \cdot \varepsilon_{i} ],\quad \left( {i = x\;{\text{or}}\;y} \right),$$

6 $$\varepsilon_{{{\text{eff}},j}} = F \cdot \varepsilon_{j} + (1 - F) \cdot \varepsilon_{1} ,\quad \left( {j = y\;{\text{or}}\;x} \right),$$

7 $$\varepsilon_{{{\text{eff}},z}} = F \cdot \varepsilon_{z} + (1 - F) \cdot \varepsilon_{1} ,$$

where *ε*_eff_ is the effective dielectric constants along different directions of the equivalent film, *ε*_*1*_ is the dielectric constant of the background of air. Note due to the optical anisotropy of the *α*-MoO_3_, *i* = *x* and *j* = *y* for the *x*-polarized wave; *i* = *y* and *j* = *x* for the *y*-polarized wave. *F* is the filling factor defined as the ratio between the area of the *α*-MoO_3_ slice and the unit area at the specific height *Z* of the *α*-MoO_3_ pyramid, and *F* can be written as: *F* = *X*^2^/*p*^2^, where *X* is the width of the *α*-MoO_3_ slice at the height of *Z*. By using the mathematical relationships of the *α*-MoO_3_ pyramid, we get a simple expression between the filling factor *F* and height *Z* as: *F* = [(*h* − *Z*)/*h*]^2^ = (1 − *Z*/*h*)^2^. Therefore, by substituting the expression of *F* into Eqs. (5)–(7), the effective dielectric constants of the equivalent film at the specific height *Z* can be obtained. By discretizing the *α*-MoO_3_ pyramid with height *h* into many equivalent thin films with thickness *δ*, the absorption responses of the equivalent film structure of the *α*-MoO_3_ SPAs can be achieved.

**Fig. 3:**
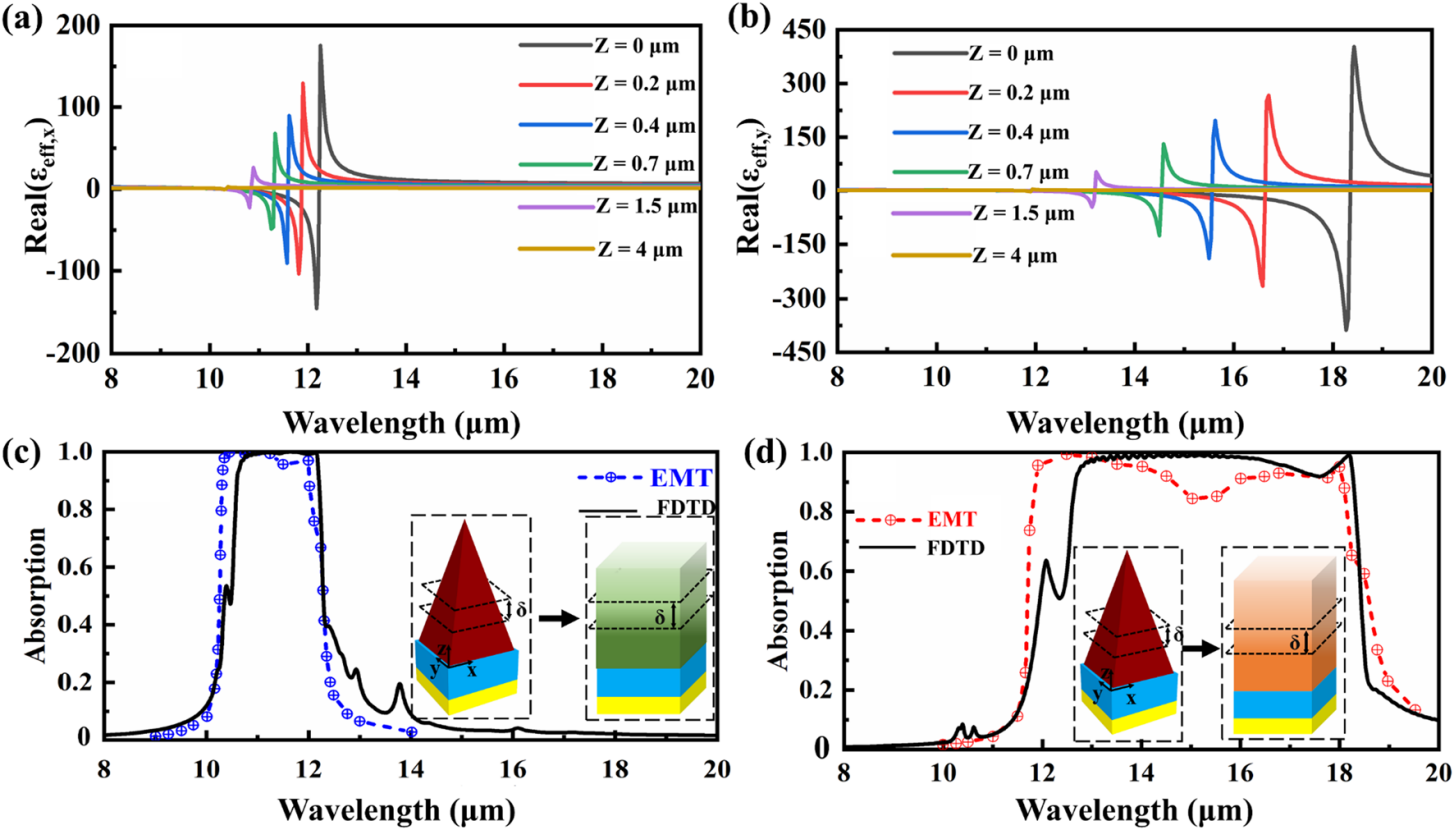
**a** Effective dielectric constant of the equivalent film for the *x* polarization as functions of locations of the height of the *α*-MoO_3_ pyramid. **b** Effective dielectric constant of the equivalent film for the *y* polarization as functions of locations of the height of the *α*-MoO_3_ pyramid. **c** Absorption responses of the equivalent film structure with *δ* = 0.1 μm and the *α*-MoO_3_ SPAs for the *x* polarization calculated by using the EMT and FDTD, respectively. **d** Absorption responses of the equivalent film structure with *δ* = 0.1 μm and the *α*-MoO_3_ SPAs for the *y* polarization calculated by using the EMT and FDTD, respectively

Figure 3 shows optical properties of the the equivalent film structure of the *α*-MoO_3_ SPAs, the schematic diagram of the equivalent processes of the two structures is inserted in the figure, and other parameters are the same as in Fig. 2a. As shown in Figs. 3a, b, for both the *x* and *y* polarizations, the dielectric constants the equivalent film structure are monotonically increased with the decrease of the height *Z*, resulting in the broadband anisotropic AR effect due to their gradient dielectric constants. In Fig. 3c, d, it can be seen that for both the *x* and *y* polarizations, the absorption responses of the equivalent film structure calculated by using EMT are agreed well with those of the *α*-MoO_3_ SPAs calculated by using FDTD, validating that the excellent selective absorption performances of the *α*-MoO_3_ SPAs are indispensable to the broadband gradient AR effects of the equivalent anisotropic film.

**Fig. 4:**
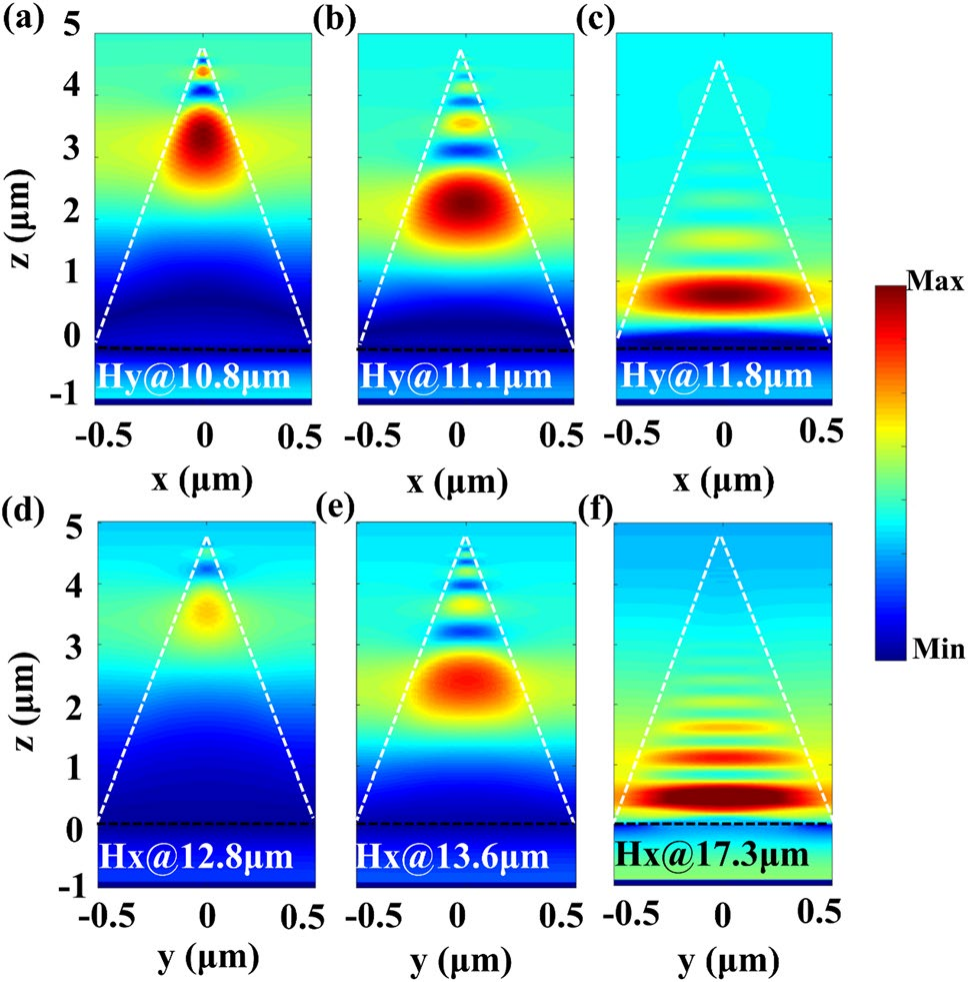
Magnetic-field distributions of band 1 for the *x* polarization at **a** 10.8 μm, **b** 11.1 μm, and **c** 11.8 μm. Magnetic-field distributions of band 2 for the *y* polarization at **d** 12.8 μm, **e** 13.6 μm, and **f** 17.3 μm. Other parameters are the same as Fig. 2a

To link the selective broadband absorption performances of the *α*-MoO_3_ SPAs with the near-field distributions of the structure, magnetic-field distributions for the *x* and *y* polarizations in both the RS band 1 and band 2 are shown in Fig. 4. As shown in Fig. 4a–c, for the *x* polarization, the light energy of the incident light can be highly trapped by the *α*-MoO_3_ pyramid due to the hyperbolic dispersion of the structure [35]. In addition, it can be found that the absorption wavelength of the resonant HPhPs mode has obviously monotonic correlation with the width of the *α*-MoO_3_ pyramid, and the lager resonant wavelength corresponds to the larger width of the *α*-MoO_3_ pyramid. This is because that the *α*-MoO_3_ pyramid can be regarded as a hyperbolic waveguide with varying width, and the field enhancement of the HPhPs mode tends to be shifted to the bottom of the *α*-MoO_3_ pyramid with the increase in the wavelength due to the lateral F–P resonance [36, 37]. In Fig. 4d–f, the resonant HPhPs mode also exhibits positive correlation with the width of the *α*-MoO_3_ pyramid in the case of the *y* polarization, and the field trapping is occurred extending from top to bottom of the *α*-MoO_3_ pyramid with the increase in absorption wavelength. Therefore, the overlapping of multiple absorption wavelengths induced by different widths of the structure leads to excellent broadband absorption of the *α*-MoO_3_ SPAs. Note with the increase in width of the *α*-MoO_3_ pyramid, the near-field coupling between the adjacent *α*-MoO_3_ pyramids results in strong field enhancement in the air gap, which contributes to broad and high absorption of the *α*-MoO_3_ SPAs.

To intuitively understand the propagation properties of the resonant HPhPs modes of the *α*-MoO_3_ SPAs, the propagation angle, and the electric-field pattern of the absorption wavelength in both RS band 1 and band 2 are investigated. Theoretically, the propagation angle of the resonant HPhPs modes of the *α*-MoO_3_ satisfies the following equation [2, 38]:

8 $$\theta_{xy} = \tan^{ - 1} \left( {\left| {\left. {\frac{{\sqrt {\varepsilon_{y} } }}{{\sqrt {\varepsilon_{x} } }}} \right|} \right.} \right),$$

where the *θ*_*xy*_ is the angle between the *x*-axis and the propagation direction of the resonant HPhPs modes. *ε*_*x*_ and *ε*_*y*_ are the corresponding dielectric constants for the excitation wavelengths of the *α*-MoO_3_, respectively. Similarly, $\theta_{yx} = \tan^{ - 1} \left( {\left| {\sqrt {\varepsilon_{x} } /\sqrt {\varepsilon_{y} } } \right|} \right)$, *θ*_*yx*_ is the angle between the *y*-axis and the propagation direction of the resonant HPhPs modes.

**Fig. 5:**
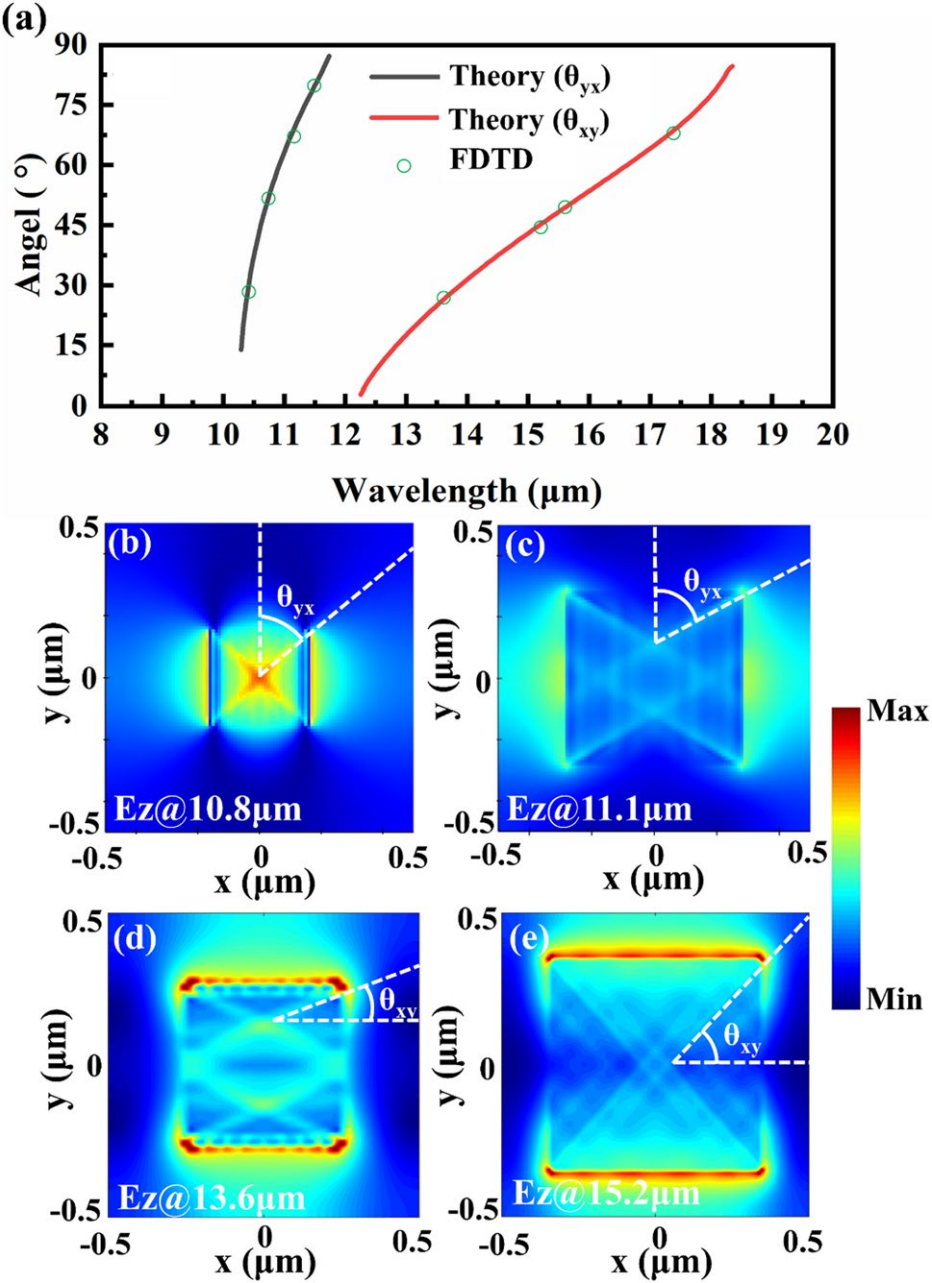
Propagation properties of the resonant HPhPs modes of the *α*-MoO_3_ SPAs, other parameters are the same as in Fig. 2a. **a** Propagation angle of the resonant HPhPs mode calculated by using theory and FDTD. Electric-field pattern **b** at 10.8 μm, and **c** at 11.1 μm in band 1 at the *xy* plane calculated by using FDTD. Electric-field pattern **d** at 13.6 μm, and **e** at 15.2 μm in band 2 at the *xy* plane calculated by using FDTD

Figure 5a shows the propagation angle of the resonant HPhPs mode calculated by using theory and FDTD in both band 1 and band 2. As shown in Fig. 5a, the theoretical results are agreed well with those of the FDTD, validating the hyperbolic propagation properties of the resonant HPhPs modes in both the two RS bands. As shown in Fig. 5b, c, the propagations of the resonant HPhPs modes for both two wavelengths in band 1 exhibit ray-like propagation features, and the theoretical propagation angles are in lined with those of the FDTD. Also, the theoretical propagation angles are agreed well with those of the FDTD for the two resonant HPhPs modes in band 2; detailed information is shown in Table 1. Note although the ray-like propagation features are not obvious at the wavelength of 15.2 μm due to the strong near-field coupling between the adjacent *α*-MoO_3_ pyramids, the theoretical propagation angle is agreed well with the FDTD result.

**Table 1:** Key parameters of the electric-field distributions in Fig. 5b–e

RS bands	Wavelength (μm)	*ε*_*x*_	*ε*_*y*_	Theory	FDTD
Band 1	10.8	− 1.97	1.23	49.5° (*θ*_*yx*_)	48.0° (*θ*_*yx*_)
	11.1	− 3.80	0.89	64.2° (*θ*_*yx*_)	61.2° (*θ*_*yx*_)
Band 2	13.6	12.64	− 3.95	29.2° (*θ*_*xy*_)	32.0° (*θ*_*xy*_)
	15.2	8.73	− 11.16	48.5° (*θ*_*xy*_)	46.7° (*θ*_*xy*_)

**Fig. 6:**
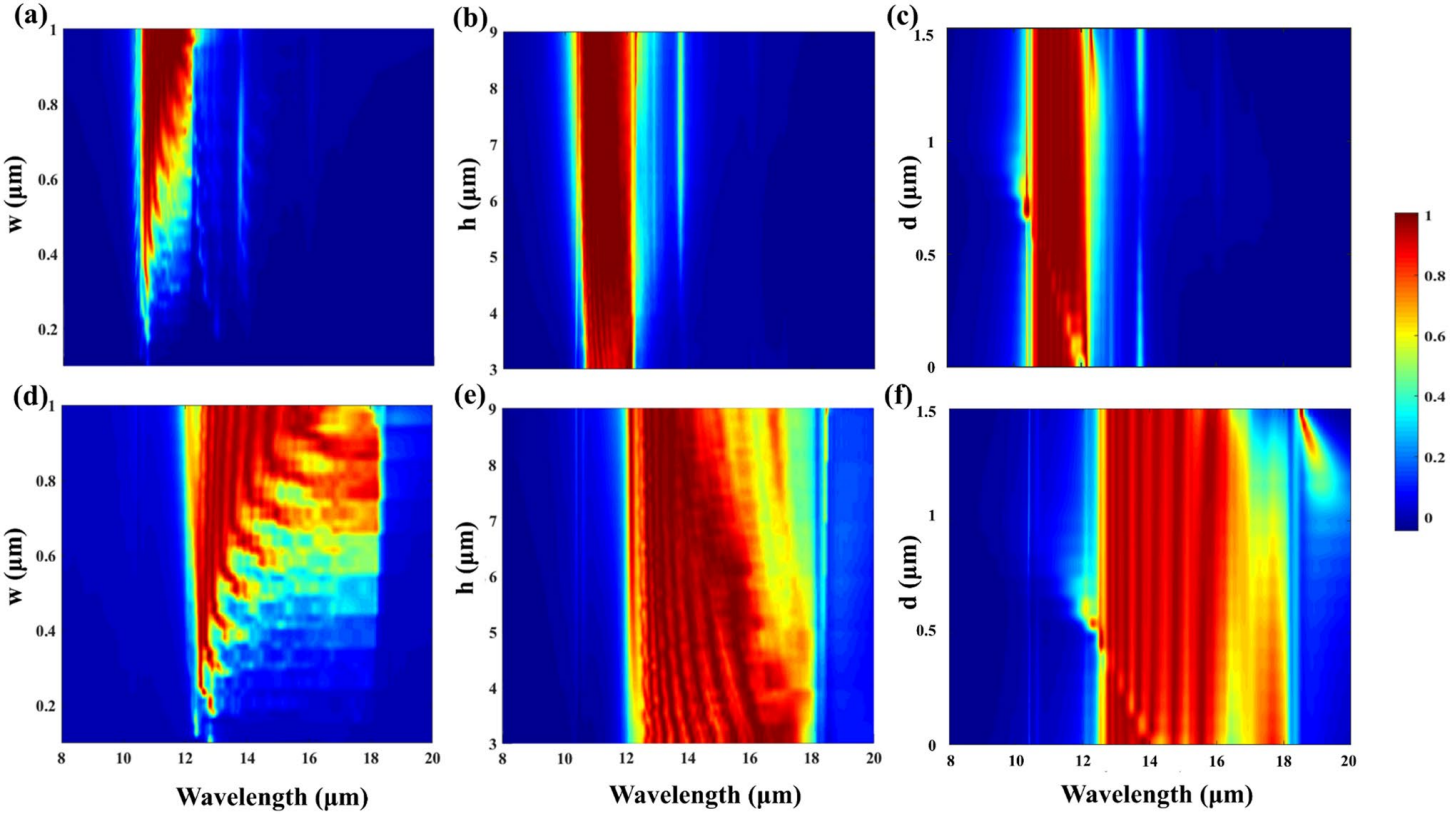
Absorption 2D map of the *α*-MoO_3_ SPAs as functions of **a** width *w* of the bottom edge, **b** height *h* of the *α*-MoO_3_ pyramid, and **c** thickness *d* of the spacer for the *x* polarization. Absorption 2D map of the *α*-MoO_3_ SPAs as functions of **d** width *w*, **e** height *h* of the *α*-MoO_3_ pyramid, and **f** thickness *d* of the spacer for the *y* polarization. Other parameters are the same as in Fig. 2a

Finally, we study the influences of the structural parameters (*w*,*h*,*d*) on the absorption performances of the SPAs for both the *x* and *y* polarizations, as shown in Fig. 6. As shown in Fig. 6a, d, the absorption performances of the *α*-MoO_3_ SPAs are sensitive to the variations of the width *w* of the bottom edge of the *α*-MoO_3_ pyramid, and broadband absorption cannot be realized if *w* is not large enough for both the *x* and *y* polarizations. However, broadband absorption can be obtained as *w* > 0.8 due to the lateral F–P resonance of the structure, and the absorption responses are robust to the variation of the *w* as well. In Figs. 6b, e, we can see that the absorption responses of the *α*-MoO_3_ SPAs are very robust to the variations of the height *h* of the *α*-MoO_3_ pyramid, and excellent selective broadband absorption can be maintained even if *h* is significantly altered for both the *x* and *y* polarizations. In addition, as shown in Figs. 6c, f, it can be seen that the broad absorption performances of the SPAs are not greatly affected by changing the thickness* d* of the spacer for both the *x* and *y* polarizations. However, the average absorption efficiency of the SPAs in the resonant bands can be improved around the optimized thickness of *d* = 0.9 μm.

## Conclusions

We have proposed and demonstrated excellent selective broadband absorption by the excitation of the resonant HPhPs modes through the *α*-MoO_3_ SPAs. For the *x* polarization, average absorption of 99.0% can be achieved within the wavelength range of 10.59–12.19 μm, and the average absorption reaches 92.5% in the whole band 1. For the *y* polarization, average absorption is as large as 97.6% within the wavelength range of 12.67–18.27 μm, and the average absorption is 89.0% in the whole band 2. For both the *x* and *y* polarizations, the absorption responses of the *α*-MoO_3_ SPAs calculated by using the EMT are agreed well with those of the FDTD, indicating the excellent selective broadband absorption of the *α*-MoO_3_ SPAs are associated with the resonant HPhPs modes assisted by the anisotropic AR effects of the structure. In addition, resonant HPhPs modes of the magnetic fields exhibit positive correlation with the width of the *α*-MoO_3_ pyramid due to the lateral F–P resonance, and the electric-field distributions exhibit the ray-like light propagation due to the resonance nature of the HPhPs modes. Finally, broadband absorption of the *α*-MoO_3_ SPAs can be maintained if the width of the bottom edge of the *α*-MoO_3_ pyramid is large than 0.8 μm, and the absorption performances are almost immune to the variations of the thickness of the spacer and the height of the *α*-MoO_3_ pyramid. We believe that the results of this study could lead to new perspectives of *α*-MoO_3_ for various applications based on its anisotropic resonant HPhPs modes.

## Data Availability

The datasets generated and/or analyzed during the current study are not publicly available due [REASON WHY DATA ARE NOT PUBLIC] but are available from the corresponding author on reasonable request.

## Abbreviations

vdW: Van der Waals
mid-IR: Mid-infrared
HPhPs: Hyperbolic phonon polaritons
SPAs: Square pyramid arrays
AR: Antireflection
EMT: Effective medium theory
F–P: Fabry–Pérot
RS bands: Reststrahlen bands
TO phonon: Transverse optical phonon
